# The impact of oral health on the quality of life of nursing home residents

**DOI:** 10.1186/s12955-015-0300-y

**Published:** 2015-07-15

**Authors:** Jessie Porter, Antiopi Ntouva, Andrew Read, Mandy Murdoch, Dennis Ola, Georgios Tsakos

**Affiliations:** UCL Research Department of Epidemiology and Public Health, University College London, 1-19 Torrington Place, London, WC1E 6BT UK; The Whittington Hospital Trust, Magdala Avenue, London, N19 5NF UK; Murdoch Health Consulting Limited, 6th Floor, New Baltic House, 65 Fenchurch Street, London, EC3M 4BE UK; Department of Clinical and Diagnostic Oral Sciences, Barts and The London School of Medicine and Dentistry, Queen Mary University of London, Turner Street, London, E1 2AD UK

**Keywords:** Older people, Oral health, Nursing home, Oral health related quality of life

## Abstract

**Background:**

Good oral health in older residents of nursing homes is important for general health and quality of life. Very few studies have assessed how oral symptoms affect residents’ quality of life.

**Objective:**

To assess the clinical and subjective oral health, including oral health related quality of life (OHRQoL), and the association of oral symptoms with OHRQoL in older people residing in nursing homes in Islington, London.

**Method:**

Overall, 325 residents from nine nursing homes were clinically examined and 180 residents were interviewed to assess their oral symptoms and their OHRQoL using the OIDP measure. Managers and carers working in the homes were also interviewed.

**Results:**

Almost two thirds of the sample were dentate (64.5 %). 61.3 % of dentate and 50.9 % of edentate residents reported problems such as dry mouth, sore cracked lips, broken teeth and toothache and ill-fitting dentures. Oral health impacted considerably upon resident’s OHRQoL; 20.2 % of dentate and 30.9 % of edentate reported at least one oral impact in the past 6 months. Sensitive teeth, toothache, bleeding gums, dry mouth and loose natural teeth among the dentate and loose or ill-fitting dentures among the edentate were strongly associated with higher prevalence of oral impacts even after adjusting for demographic and socio-economic factors, and for the number of teeth (dentate only).

**Conclusion:**

The burden of oral conditions was considerable. Oral symptoms were very common and were strongly associated with residents’ worse OHRQoL. Health promotion programmes are important to help residents maintain an acceptable level of oral health and function.

## Background

The ageing of the population brings new challenges for oral health. As older people live longer and retain more of their natural teeth than previous generations, they are more likely to be more functionally dependent [1]. This is expected to increase dental treatment needs and place more demand on dental and health care systems [2].

Oral health has a significant impact on general health especially among older adults, as the ability to chew influences patterns of food consumption and diet quality [3–5]. Poor diet has been associated with impairment in cognition and function, which in the elderly may add to the burden of age-related cognitive decline [6–8]. Poor oral hygiene, tooth loss and diseases from oral pathogens have been linked with other non-communicable diseases such as diabetes, pneumonia and circulatory diseases [9–12]. In particular, the importance of oral disease and the occurrence of aspirational pneumonia has been conclusively demonstrated in frail elderly patients and has been suggested that tooth brushing, denture cleaning and professional oral health care may have beneficial impact on reducing the incidence of aspirational pneumonia [12]. Therefore, poor oral health can have a significant impact on the quality of life and the ability of an individual to go about their daily routines [13, 14].

Elderly people living in nursing homes are a particularly vulnerable group as they have poorer oral health than the general population of older adults [15–18]. Due to varying degrees of physical and cognitive decline, and related behavioural issues, residents may rely heavily on carers for all aspects of their oral care, relinquishing control and personal autonomy. Oral health in nursing homes is often not seen as a priority, and this could potentially delay the assessment, identification and treatment of dental problems of the residents [19–21].

Most studies on the health of nursing home residents report that their oral health status is poor [16, 17, 22, 23]. Studies on residents’ oral health and treatment needs have mostly used clinical examinations and without exploring how residents perceive their oral health and the impact it has on their quality of life. In the few instances where questionnaires were used for data collection, the emphasis was mainly on oral health behaviours and dental attendance, without assessing the impact of oral conditions on the residents’ quality of life [16, 17, 24].. A more comprehensive view of the oral health and quality of life of people living in nursing homes can be obtained by looking at clinical status and at subjective aspects of oral health and oral health related quality of life of the residents, as well as sampling the views of care home managers and staff. [16, 24, 25] Therefore, this study aimed to assess the clinical and subjective oral health, including oral health related quality of life (OHRQoL), and the association of oral symptoms with OHRQoL in a sample of older people residing in nursing homes in Islington, London. Furthermore, we also assessed the views of nursing home staff and managers regarding the oral health status and care of the residents.

## Methods

This was a cross-sectional epidemiological survey of residents, carers and managers of all nine nursing homes in Islington. The nine nursing homes ranged in size from 29 to 87 beds and 22 to 79 residents. Islington is a relatively deprived London borough [26], the fifth most deprived in London, with 39 % of over 60’s in Islington receiving pension credit compared to 24 % in London [27] We initially contacted the managers of the nursing homes to explain the purpose and content of the study and request permission to carry it out. In collaboration with the nursing homes staff, appointments were scheduled for the residents’ clinical examinations and the completion of questionnaires. In addition, managers and carers in the nursing homes were approached to schedule appointments for interviews. An earlier study had been carried out in the same nursing homes in 2009 [28]. Since then, as a result of the recommendations in the 2009 survey, these nursing homes have introduced an oral health improvement plan that included a basic oral health assessment and oral health promotion activities through oral hygiene, referrals for dental care and staff training.

Ethical approval for the study was gained from the UCL Ethics Committee (REF:2000/002).

### Data collection

Data from the residents was collected through a clinical oral examination and an interviewer-administered questionnaire. For managers and carers, data collection was based on an interviewer-administered questionnaire.

All residents aged 65 to 100 years were invited to participate. Eligibility for the interviewer-administered questionnaire was based on passing a simple cognitive test, consisting of four questions, such as ‘what is your name’ ‘on what date were you born’ ‘do you remember what year it is now’ ‘do you recall the name of the present queen’. An information sheet, explaining the purpose of the survey and the procedures involved in participating, and declaring confidentiality of the data and anonymity of subjects, was distributed to the 448 registered residents in the nine homes, along with a consent form to participate in the study. If a resident was unable to give verbal or written consent, consent was obtained from the resident’s next of kin.

The managers and carers invited to participate in the study were provided with a relevant information sheet and asked for verbal consent. All nine managers were interviewed. In addition, one nurse and three carers were randomly selected from each home to participate in the study, a total of 36 participated in the study.

A non-invasive clinical examination was carried out by a dentist using a mirror and a blunt ended probe (for removal of debris), and cotton wool rolls. The clinical examination assessed the condition of crowns and roots, spacing and occlusion, as well as soft tissues and conditions relating to partial and complete dentures. The clinical examination criteria were adapted from the National Diet and Nutrition Survey (NDNS) of older adults [29]. Caries were determined according to visual detection or presence of cavity in the dentine and did not include “arrested” caries or stained fissures (unless they also had evidence of caries). Infection control was adhered to according to British Dental Association Cross infection guidance [30].

An oral health promoter collected questionnaire data through face-to-face interviews with the residents, managers, carers and nurses. The residents’ questionnaire covered the demographic profile (age, sex, ethnicity), socioeconomic position (education level, pension status), oral symptoms (sensitive teeth, toothache, broken teeth, missing or loose teeth, bleeding gums, dry mouth, burning sensation in the mouth and dry, sore or cracked lips) and OHRQoL. For this, the Oral Impacts on Daily Performances (OIDP) measure [31] was used to assess the impact of oral conditions on the daily life of the residents through the following items: difficulty eating, difficulty speaking, difficulty cleaning teeth or dentures, difficulty relaxing (including sleeping), problems smiling, laughing or showing teeth without embarrassment, emotional problems such as becoming more easily upset, and problems enjoying the contact of other people such as relatives and friends. In its full version, the OIDP assesses the frequency and severity of each oral impact. However, in order to reduce respondent burden in our study, we only asked about the presence of each of these impacts. This allowed us to calculate the overall prevalence and also the prevalence of each one of these oral impacts, but we cannot provide the OIDP score.

For the managers and the carers, the respective questionnaires included questions on number of residents and staff, access of residents to dental services, oral assessment of residents by nursing home staff, assessment of education and training of staff, facilities and support for personal oral hygiene and any help nursing homes may need from the NHS relating to oral health.

### Data management and analysis

Data were entered in an Excel file and checked for logical inconsistencies. Data analysis was carried out using Stata™ version 12.0 (STATA Corp, Texas, USA) and referred to quantitative analysis of residents’ clinical examinations and interviews. Initial descriptive statistics presented the distinction of the sample between dentate and edentate and the clinical profile for each of these two different groups. Prevalence was calculated for binary variables, such as presence of dental decay, and mean (together with standard deviation) was presented for count variables, such as number of decayed teeth. In addition to the analysis for the whole dentate or edentate sample, we also presented estimates (mean and standard deviation) for count clinical variables among those with a specific condition; for example, mean and standard deviation of decayed teeth among those with tooth decay. This would show the burden of disease among those suffering from it and complements the overall picture from the whole sample that also includes many residents without the specific oral condition.

For residents that participated in the interview, we also calculated the prevalence of different oral health problems; sensitive teeth, toothache, broken or chipped teeth, loose natural tooth, loose or ill-fitting denture, bleeding gums, dryness in the mouth, burning sensation in the mouth or dry, sore or cracked lips. For OHRQoL, we presented prevalence estimates for the overall prevalence of oral impacts and of each OIDP item. Then, multivariable logistic regression models assessed the relationship between oral problems and oral health related quality of life. For these models, the prevalence of oral impacts (at least one OIDP item with a non-0 score) was the outcome, while five different oral problems (sensitive teeth, toothache, loose natural teeth, bleeding gums and dry mouth) were alternately used as the main exposure variable. The initial models adjusted for the effect of demographics (age and sex) and education level, and the fully adjusted model also controlled for clinical oral health (number of teeth). Similar analysis was also carried out for loose or ill-fitting dentures for the edentate residents.

For the data collected through the interviews with managers and carers, simple frequency distributions highlighted the key themes identified and these are presented narratively in the Results.

## Results

### Carers and managers questionnaire

Of the nine nursing homes, three were privately owned and managed, one run by a charity organisation, one by the local authority and four were private, but managed jointly with the local authority. In general, the carers and managers expressed similar views and the key findings are therefore presented together.

Almost all interviewed staff reported that they were aware of the Care Quality Commission standards that relate to the oral health of the nursing home residents and that an oral health assessment was made for all new residents, in most cases within a week of their arrival to the nursing home. In addition, these assessments were made by staff trained for that purpose by oral health promoters. Furthermore, this initial assessment was in most homes repeated yearly and it covered a wide range of issues, from an overall assessment of the number of teeth present in the mouth, whether they wore dentures or not, an overall assessment of the soft tissues in the mouth and the current problems with oral health (e.g. toothache), while also extending to behavioural risk factors for oral health (e.g. oral hygiene, diet) and finally covering oral impacts such as the difficulty in eating and chewing food. However, the interviews raised also a number of key concerns in relation to the oral health and dental care of the residents.

One of the main oral health concerns that the staff in nursing homes had relate to the residents’ ill-fitting or loose dentures. They felt that a large proportion of the residents that wore dentures had difficulty with their retention and this was a recurrent source of complaint. In addition, there were quite a few comments that related to “gum problems” and a number of the staff mentioned that residents tended to complain about those irrespective of whether they had their natural teeth or wore dentures. Possibly in line with the above, staff also reported that a high proportion of their residents had difficulty chewing and eating hard foods.

Another area of concern referred to oral hygiene practices. Many staff members reported that they faced difficulties in terms of the residents’ behavioural issues relating to oral health, such as their refusal to cooperate with the suggested oral hygiene regime or even their denial to open their mouth so that the carers could help them brush their teeth. The issue of availability and accessibility of dental services was also mentioned here. Five out of nine managers reported that there was a need to train the staff to appropriately refer residents to receive dental care and a number of staff also reported the difficulties that some of the residents faced in that respect.

Finally, all managers and the vast majority of the staff reported issues surrounding dementia as a large number of nursing home residents were reported to be suffering from it. This concern is related to both aforementioned problems with oral hygiene practices and dental care arrangements. For example, it was mentioned that while it was possible to arrange dental appointments the residents may have changed their mind by the time of the appointment and they mentioned the dementia as a potential reason for this.

### Residents

From the 448 residents in the nine homes, 45 were not eligible (outside the chosen age range of 65–100 years). Of the 403 eligible residents, 72 were not included in the sample because on the day of the survey they were either too fragile or agitated to participate, or were attending a hospital. A total of 331 residents participated, a response rate of 82 %. The clinical oral examination was conducted on 325 residents because six participants refused to undertake that part of the survey. For the interviewer-administered questionnaire, 151 residents failed the cognitive examination and were excluded, resulting therefore on 180 residents for that part of the survey.

The majority of the participating residents were female (64.8 %). Their mean age was 82.2 [sd: 7.64] years; 64.4 % identified themselves as White British. For socioeconomic position, 44.1 % of the residents that answered the questionnaire received the state pension only, while the sample was spread across education level groups; 33.2 % only finished primary school, 37.1 % secondary school and 29.8 % finished College/University.

Of the 325 clinically examined residents, 202 (62.2 %) were dentate, with a mean number of 18.0 [sd: 8.3] teeth missing and 123 (37.8 %) were edentate. Among the edentate, only 40.7 % wore a denture. Overall, 41.1 % and 64.9 % of the dentate residents had coronal and root caries respectively. There was an average of 2.0 [sd: 1.6] decayed teeth among those that had active tooth decay (*n* = 84), and the respective figure for decay among those with decayed roots (*n* = 131) was 3.4 [sd: 2.8] roots. As expected, almost all (99.5 %) residents had experienced tooth loss with an average of 18.1 [sd: 8.3] missing teeth, while among those with restorations (*n* = 137) there were on average 6.2 [sd: 5.1] filled teeth. Among those with tooth mobility (*n* = 63), there were on average 2.7 [sd: 2.3] teeth that were mobile, while those with unfilled anterior spaces (*n* = 139) had on average 5.8 [sd: 5.1] spaces. The clinical status of the dentate residents can be seen in Table 1.

**Table 1:** Clinical dental status of dentate residents (*n* = 202)

Clinical status	Percentage	Mean (S.D.) among the whole sample	Mean (S.D.) among those with the specific condition
	(number)		
Sound teeth	5.0 % (10)	8.9 (6.37)	9.38 (6.19)
Decayed teeth	41.1 % (83)	2.0 (1.64)	1.97 (2.06)
Filled teeth	67.8 % (137)	4.2 (5.13)	6.21 (5.15)
Missing teeth	99.5 % (201)	18.0 (8.28)	18.12 (8.20)
Decayed roots	64.9 % (131)	2.2 (2.77)	3.40 (2.79)
Tooth mobility	31.2 % (63)	0.9 (1.79)	2.74 (2.29)
Unfilled anterior space	68.8 % (139)	4.0 (5.04)	5.86 (5.12)

In terms of subjectively reported oral problems (Table 2), over half of dentate (61.3 %) and edentate (50.9 %) residents reported at least one problem. As expected, many edentate residents experienced problems with dentures, with 34.4 % suffering from loose or ill-fitting dentures, along with 4.3 % of dentate residents. Dryness of the mouth was prevalent for both dentate (41.2 %) and edentate (40.0 %) residents, along with dry, sore or cracked lips (33.6 % in dentate and 38.2 % in edentate residents). In the dentate residents, many experienced issues such as broken teeth (23.9 %), toothache (17.1 %) and sensitivity (15.4 %).

**Table 2:** Self-rated oral health problems, by dentate (*n* = 124) and edentate (*n* = 55) residents interviewed

Oral problem	Dentate	Edentate
	(*n* = 124)	(*n* = 55)
Sensitive teeth	15.4 %	N/A
Toothache or severe discomfort	17.1 %	N/A
Broken tooth	23.9 %	N/A
Loose natural tooth	15.4 %	N/A
Loose or ill-fitting denture	4.3 %	34.4 %
Bleeding gum	10.1 %	N/A
Dryness of the mouth	41.2 %	40.0 %
Burning sensation in the mouth	3.4 %	3.6 %
Dry, sore or cracked lip	33.6 %	38.2 %
Any problem	61.3 %	50.9 %

Table 3 indicates the impact oral conditions had on the everyday lives of residents. Overall, 20.2 % of dentate and 30.9 % of edentate reported at least one oral impact in the past 6 months. Difficulty eating (29.1 % of edentate and 16.9 % of dentate) was the most prevalent oral impact, followed by difficulty speaking (6.5 % of dentate and 16.4 % of edentate). Problems smiling or laughing or showing teeth without embarrassment were also reported by 4.0 % of dentate and 9.1 % of edentate residents. Oral conditions affected emotionally 1.6 % of dentate and 5.5 % of edentate residents, while the respective estimates for an oral impact on not being able to enjoy contact with other people were 3.2 and 3.6 %.

**Table 3:** Impact of oral conditions on daily activities, by dentate (*n* = 124) and edentate (n = 55) residents interviewed

Impact	Dentate (*n* = 124)	Edentate (*n* = 55)
Difficulty eating	16.9 %	29.1 %
Difficulty speaking	6.5 %	16.4 %
Difficulty cleaning teeth/denture	3.2 %	0 %
Difficulty relaxing or sleeping	0.8 %	0 %
Problems smiling or laughing or showing teeth without embarrassment	4.0 %	9.1 %
Emotional problems	1.6 %	5.5 %
Cannot enjoy contact with other people	3.2 %	3.6 %
Any impact	20.2 %	30.9 %

Perceived oral problems were significantly associated with OHRQoL among dentate residents (Table 4). Residents suffering from sensitive teeth were 4.32 (1.30, 14.38) times more likely to also report at least one oral impact (OIDP > 0) compared to those without sensitive teeth in the fully adjusted model (i.e. adjusting for demographic factors, education level and clinical status). Toothache was associated with much higher odds of also reporting oral impacts; even after adjusting for demographics (age, sex), education level and clinical status (number of teeth), residents that experienced toothache were 10.86 (2.88, 40.96) times more likely to report at least one oral impact on their daily life in the past 6 months compared to those without toothache. Strong associations with worse OHRQoL were also shown for having a loose natural tooth, bleeding gums, and dry mouth. In the fully adjusted models, residents who had loose natural teeth were 5.32 (1.70, 16.60) times more likely to report at least one oral impact compared to those without loose teeth, while the respective odds ratio for those reporting bleeding gums was 8.70 (2.05, 36.92). Similarly, residents that reported dry mouth were 3.91 (1.46, 10.46) times more likely to also report at least one oral impact than their counterparts without dry mouth (Table 4).

**Table 4:** Association between self-reported oral problems and oral health related quality of life among dentate residents (*n* = 116)^a^

	At least one oral health impact (OIDP > 0)			
	Age-Sex-Education adjusted model		Age-Sex-Education-clinical status^b^ adjusted model	
	Odds Ratio (95 % CI)	*P* value	Odds Ratio (95 % CI)	*P* value
Dentate				
Sensitive teeth	4.24 (1.29 to 13.89)	0.017	4.32 (1.30 to 14.38)	0.017
Toothache	10.66 (2.97 to 38.17)	<0.001	10.86 (2.88 to 40.96)	<0.001
Loose natural teeth	5.32 (1.70 to 16.60)	0.004	5.74 (1.77 to 18.59)	0.004
Bleeding gums	8.30 (2.06 to 33.47)	0.003	8.70 (2.05 to 36.92)	0.003
Dry mouth	3.74 (1.42 to 9.87)	0.008	3.91 (1.46 to 10.46)	0.007

^a^Overall, 124 dentate residents responded to the questionnaire, but 8 did not provide data on oral health problems, therefore *n* = 116 for this analysis

^b^Measure of clinical status in this model was number of natural teeth

Those with loose or ill-fitting dentures were 9.07 (1.54, 55.55) times more likely to report at least one oral impact than those with well-fitting dentures, after adjusting for demographic factors and education level.

## Discussion

This study documented the oral health problems in a sample of nursing home elderly residents in an inner London borough and showed that they have a considerable impact on the quality of life of the residents. The burden of oral conditions of the residents was considerable. More than one third were edentate but only about 40 % of them wore a denture. Tooth loss and tooth and root decay were common among the dentate. Over half of dentate and edentate residents reported oral health related problems, predominantly dry mouth and ill-fitting dentures; the prevalence of those conditions was high even for quite extreme symptoms. For example, 17.1 % of the dentate residents reported toothache in the past 6 months. Oral health related problems had a profound impact on their quality of life; 20.2 % of dentate and 30.9 % of edentate reported at least one impact on their daily life.

More importantly, oral problems, and in particular sensitive teeth, toothache, bleeding gums, dry mouth and loose natural teeth among the dentate and loose or ill-fitting dentures among the edentate, were strongly associated with worse OHRQoL, i.e. with a higher prevalence of oral impacts. And the respective associations were strong and remained so irrespective of the effect of demographic and socio-economic factors (for both dentate and edentate) and number of teeth present in the mouth (for the dentate). That indicates that OHRQoL was strongly affected mainly by oral symptoms and problems.

The overall findings are comparable with other studies, namely, that oral health in nursing home residents is poor [8, 17, 18, 22, 23, 32]. Additionally, this study also found that residents felt their oral health impacted on their quality of life quite significantly, as reported in two similar studies [33, 34]. Previous studies have rarely interviewed the residents themselves. When they were, questionnaires only focused on oral health behaviours without looking at OHRQoL [16, 24, 25]. Quality of life is an important outcome, especially in an elderly population, as clinical indicators are not always sufficient to describe an individual’s health status, with poor general health not necessarily resulting in poor quality of life. There are numerous studies reporting the impact of oral health on quality of life of older people [35, 36]. However, they were carried out on free living populations; very few focused on people living in nursing homes [37, 38]. One was an early study on 58 nursing home residents in New York [38], while the other was carried out in Toronto, Canada, and had a detailed questionnaire primarily around dry mouth and OHRQoL, but no clinical examination [37]. In line with parts of our results, this study demonstrated the importance influence of dry mouth on the quality of life of nursing home residents.

Since the earlier survey in 2009, the nursing homes have embarked on an oral health improvement programme which included oral health assessments of new residents, residents being assisted to carry out their daily oral hygiene routine, and improvement of dental services referral pathways. Furthermore, the nursing homes staff have been trained to undertake all these duties. Looking at the overall picture of oral health and quality of life in the 2009 survey and the results presented here, it seems that the oral health **and related quality of life** of the Islington nursing home residents **is currently better than what it was** in the 2009 survey. Our results showed **a higher proportion of dentate residents (56.9 % in 2009, 62.2 % in 2013)**. Among the dentate, tooth mobility was less common **(38.5 % in 2009, 31.1 % in 2013)**, there were more restored teeth **(a mean of 3.0 teeth in 2009 and 4.2 in 2013)** and there was also a marked increase in dental residents being seen in the Community Dental Service, from 36 to 80 %. More importantly, the prevalence of toothache **or severe discomfort** was much lower **(24.2 % in 2009 and 17.1 % in 2013)** and the residents reported markedly better ratings in terms of their OHRQoL **(in 2009, 36.6 % of dentate and 43.0 % of edentate reported oral impacts, while the respective figures in 2013 were 20.2 and 30.9 %)**.

The managers and carers interviews have helped to highlight some key issues in relation to the oral health practices in nursing homes. Overall, managers understand the importance of oral health and there seems to be a good level of awareness of the relevant CQC monitoring standards and in all cases there were procedures established in order to make sure that the oral health of the residents was not neglected. However, there are still some important concerns in relation to the oral health of the residents, particularly in terms of dealing with cooperation with the staff for their oral hygiene practices and also with access to dental services.

Our results have some direct public health and service implications for the oral health of the nursing homes residents. We acknowledge that the provision of dental care (preventive and treatment) services in nursing homes may be difficult. Residents can be physically dependent, cognitively compromised, suffering from multi-morbidity and having many complex medical needs. Despite these challenges, the high levels of need and impacts of oral health on the quality of life of the residents highlight the importance of broader health promotion interventions that go some way towards helping residents maintain an acceptable level of oral health and oral functioning. Furthermore, the high prevalence of ill-fitting dentures implies that the provision and repair of dentures should be the main focus of dental treatment provision through intensifying referral procedures. In addition, our results indicate the importance of more oral health training and education of nurses and carers so that they can help the residents maintain decent oral health and address their basic dental care needs. These programmes should focus on training and equipping nursing home staff with the knowledge and skills to support nursing home residents, as well as improving access to dental services. Health promotion programmes are important to help residents maintain an acceptable level of oral health and function.

This was an epidemiological survey and the clinical examinations were carried out in the nursing home with basic equipment and under field conditions, rather than in a dental surgery with the use of extensive diagnostic tools. This may have underestimated the extent of oral diseases and dental treatment needs. We also note that approximately 40 % of the edentate residents did not wear a denture. Future studies should also establish to what extent this was due to the barriers to access relevant dental care or due to other reasons. Furthermore, oral problems/symptoms and oral impacts are subjective outcomes and sometimes participants, particularly the elderly, may be unable to recall their experiences fully, while response bias due to social desirability can also affect the quality of the data. However, we have attempted to minimise any such bias by employing a widely used and validated OHRQoL measure and training the interviewers. The inclusion of perceptions about oral health and related quality of life is a strength of our study as it gives a more comprehensive picture of oral health and its impacts on this vulnerable population. Self-report measures are important in oral health as they go beyond the historical disease-based clinical measures and highlight the psychological and social consequences of oral disorders. However, we do acknowledge that had we used the full version of the OIDP we would be able to provide information not on oy on the prevalence but also on the severity of oral impacts.

## Conclusions

The burden of oral conditions was considerable among nursing home residents in an inner London borough. The main clinical issues related to the quality of dentures as well as to the high prevalence of tooth and root decay. Oral symptoms, such as dry mouth and toothache, were also very common and they were strongly associated with the residents’ worse OHRQoL.
